# Characterizing Elder Abuse in the UK: A Description of Cases Reported to a National Helpline

**DOI:** 10.1177/07334648221109513

**Published:** 2022-06-28

**Authors:** Silvia Fraga Dominguez, Jennifer E. Storey, Emily Glorney

**Affiliations:** 1 1725 Birmingham City University, UK; 2 Royal Holloway, 3162 University of London, UK; 3 2240 University of Kent, UK

**Keywords:** elder mistreatment, case characteristics, helpline, elder neglect, poly-victimization, older adults

## Abstract

The abuse of older adults by someone in a position of trust—also known as elder abuse (EA)—has a severe impact on victims and society. However, knowledge about EA in the UK is limited in comparison to other types of interpersonal violence and international knowledge. The present study utilized secondary data from a UK national EA helpline to investigate the characteristics of reported cases. Over a one-year period between 2017 and 2018, 1,623 records met inclusion criteria. Descriptive statistics are provided to describe this sample. Most cases reported to the helpline pertained to female victims, suffering from financial or psychological abuse. Co-occurrence of different abuse types was common. Findings provide updated knowledge about the phenomenology of EA cases in the UK. Recommendations are provided for advancing research in this area, including the need for examining cases across longer periods of time with a view to informing practice and policy.

**What this paper adds**

• This paper represents the description of the largest sample of EA cases in the UK and updates existing knowledge about abuse against older adults in the country, where research on the topic is limited as compared to countries like the United States and in relation to other types of interpersonal violence.
• It helps to understand the complexity of EA, by including cases of abuse occurring in institutions and the community, and cases in which victims may not be able to self-advocate.

**Applications of study findings**

• The findings, which contrast with previous findings in the UK, underscore the need to study EA using different sources of data (prevalence, helpline records, and police/safeguarding records)—as each data source is biased in different ways and represents a different group of victims, perpetrators, and abuse types.
• The findings also suggest the need to study EA across longer periods of time—to understand changes in the nature of EA as demographics evolve.
• This study suggests that financial, psychological abuse, and poly-victimization are priorities in terms of research, prevention, and intervention.

Elder abuse (EA) (also known as elder mistreatment and older adult abuse/mistreatment) is “a single or repeated act, or lack of appropriate action, occurring within any relationship where there is an expectation of trust, which causes harm or distress to an older person” (World Health Organization [WHO], 2021, para. 2). Elder abuse is prevalent worldwide, estimated to affect one in six older adults in the community according to a recent meta-analysis (Yon et al., 2017) and is also perpetrated in institutions such as care homes, with 64% of staff self-reporting mistreatment (see Yon et al., 2018). There are different EA types recognized: financial abuse/exploitation, physical, sexual, psychological or emotional abuse, and neglect (Pillemer et al., 2016), with abuse types known to co-occur (Brijnath et al., 2021; Jackson & Hafemeister, 2011; Lachs & Berman, 2011; Ramsey-Klawsnik, 2017; Weissberger et al., 2020; Williams et al., 2020). Elder abuse has been linked to severe consequences for victims such as psychological and physical harm and financial loss, with poly-victimization—the co-occurrence of abuse types—likely to result in increased impact (Jackson & Hafemeister, 2011; Ramsey-Klawsnik, 2017; Yunus et al., 2019).

Despite its prevalence and impact, EA has been identified as the most overlooked type of interpersonal violence, receiving far less attention than intimate partner violence and child maltreatment (Butchart & Mikton, 2014; Dyer et al., 2003). Further, research advances are not uniform, and there is a large gap between EA research in the United States, where more than half of the studies have been published, and in other countries (Sweileh, 2021). In the United Kingdom (UK), EA research is understood to lag 10 years in comparison with the United States (Penhale & Kingston, 1997) and the latest nationwide prevalence study was conducted more than 15 years ago (O’Keeffe et al., 2007). Researchers estimated then that EA affected between 2.6% and 4% of adults 66 years and older living in private households—with acquaintances and neighbors included as perpetrators in the second figure (O’Keeffe et al., 2007). The researchers found that victims were more often women than men, and that neglect and financial abuse were the most prevalent. The most common perpetrator was the victim’s partner/spouse, followed by other relatives (O’Keeffe et al., 2007).

Aside from prevalence studies, other sources of data can provide information about EA cases, including reports to formal organizations that may initiate an investigation (e.g., the police or adult safeguarding) or to national helplines offering advice (Weissberger et al., 2020). Police or adult safeguarding records may under-estimate the true extent of the problem, given that only between 4 and 14% of cases are estimated to reach formal response systems (Lachs & Berman, 2011; Weissberger et al., 2020). In addition, uniform reporting systems are not available in every country, including the UK. Conversely, helpline records may capture cases that are never reported to criminal justice or adult safeguarding professionals, where older adults and other third parties are seeking advice without requesting an investigation (Weissberger et al., 2020). Studies based on these data have recently been published in the United States (Weissberger et al., 2020) and Australia (Brijnath et al., 2021) and have highlighted their strengths in offering information about the nature of the cases in a country, particularly if the helpline is nationally available and free to access. For example, helpline data can encompass both community and institutional cases of abuse, while prevalence studies are usually conducted in one of these two settings (Bennett et al., 2000; O’Keeffe et al., 2007; Yon et al., 2017, 2018).

To the authors’ knowledge, the most recent analysis of cases reported to a helpline in the UK was published more than 20 years ago (Bennett et al., 2000). The study authors provided a descriptive analysis of 1,421 EA-related calls made over a 2-year period to the helpline of a national charity called Action on Elder Abuse. Callers were often third parties, particularly relatives, and they detailed abuse happening at the victim’s home, primarily of psychological nature, but also frequently financial or physical, and perpetrated by a relative (Bennett et al., 2000). Victims were often female, with a more equal gender divide for perpetrators, although male perpetrators were slightly more common, consistent with recent research in Australia (Brijnath et al., 2021). Information about the characteristics of victims and perpetrators (e.g., vulnerability and risk factors), as well as further details of the abuse, and how often specific abuse types (e.g., financial and neglect) co-occurred was not reported. Because of the age and lack of depth of these data, they are limited as to how much they can inform current approaches to responding to EA cases in the UK.

Currently, the UK lacks a recent overview of the characteristics of EA cases in the country, given that both prevalence studies and those based on helpline data are outdated. In addition, many of the variables that have grown in importance in recent research, such as victim-perpetrator relationship dynamics or specific abusive behaviors, have not been fully explored. Although the prevalence study provided a more detailed picture than the helpline publication, the former did not include cases of victims with dementia or institutional abuse cases; thus, limiting representativeness. In addition, in the prevalence data from 2007, neither of the participants had reported to a helpline, implying that these cases have not been recently represented in research (O’Keeffe et al., 2007). Updated knowledge about EA is necessary to tailor policies and intervention approaches to the nature and dynamics of EA (Brijnath et al., 2021), particularly considering substantial demographic changes in terms of the ageing of the population (WHO, 2021). The advantages of using secondary data from a helpline are that it may represent cases that are not encompassed in surveys or in reports to the police or adult safeguarding, as well as EA in both the community and institutions (Weissberger et al., 2020). Furthermore, a cross-sectional exploration of EA over a defined period offers an opportunity to capture breadth and context of EA concerns and victimization.

## The Present Study

The present study aims to provide updated knowledge by describing the characteristics of cases reported to a national free helpline. The authors focused on the characteristics of (a) the enquiry, (b) the alleged victim and perpetrator, (c) their relationship, and (d) the abuse. Variables within these categories were gathered as part of a larger project with the purpose of studying these in relation to help-seeking behaviors by victims and informal third parties (see Fraga Dominguez et al., 2021a). Throughout this paper, those alleged to have engaged in EA will be referred to as perpetrators and those who were allegedly subjected to abuse will be referred to as victims.

## Design and Methodology

### Research Design and Data Source

This study involved a secondary analysis of cross-sectional data focused on understanding help-seeking in EA. The data encompassed all the records entered in an EA helpline’s dataset between 22/5/2017 and 22/5/2018. Some records could be linked to a previous contact with the helpline, which could have occurred during the target period. For example, a record in April 2018 could be subsequent to a previous enquiry made in August 2017. In these cases, the information from any further enquiry (in the example, April 2018) was added to the information about the first record (in the example, August 2017) of that same case in the dataset.

The data source was Hourglass’ free helpline. Hourglass, formerly called Action on Elder Abuse and founded in 1993, is the only EA-dedicated charity in the UK (Hourglass, n. d.; O’Keeffe et al., 2007; Podnieks et al., 2010). Recently, the organization has broadened their focus to include the general promotion of safer aging (Hourglass, n. d.). At the time of the study, Hourglass’ helpline, which has been operational since 1998, operated from Monday to Friday during working hours (Action on Elder Abuse, 2008; Bennett et al., 2000). Cases have been recorded and managed electronically since 2017. The main objective of the helpline is to offer advice to people suffering from EA and others (e.g., relatives, friends, and professionals) who are seeking guidance on behalf of EA victims, and signpost them to appropriate services. The helpline is supported by trained staff and volunteers, and, at the time of conducting the study, the public could contact them via telephone, email, or letter. When a helpline worker/volunteer received an enquiry, they recorded a free text describing the enquirer’s situation, the help needed, and the advice provided. Following these free texts, workers/volunteers filled out fields with some basic information about the enquiry (e.g., victim’s age).

### Procedure

The first author received access to the helpline records for the purpose of the study through a written agreement and signed a confidentiality agreement with the charity. The researchers received ethical approval from their institution on 7^th^ May 2018.

The focus of the study were all enquiries (i.e., calls, emails, and letters) during the period from May 2017 to May 2018 (*N* = 2,538). Data coding started in October 2018 and was completed in May 2019. Prior to coding the data, the researchers developed inclusion criteria, with the objective of including EA cases with sufficient information to describe the sample and answer research questions related to help-seeking. The case had to:

1) Be considered EA, as understood by the charity Hourglass and the WHO (2021), which uses the definition coined by said charity (Action on Elder Abuse, 1995). To guide decision-making in cases with more limited information, attention was paid to the helpline’s recommended actions (e.g., whether they recommended an EA organization or otherwise indicated that the case did not constitute EA). This procedure is consistent with a recent study in the United States focused on helpline enquiries (Weissberger et al., 2020). The age cut-off was 60, in accordance with the WHO (2021) and previous research (Brijnath et al., 2021; Weissberger et al., 2020; Yon et al., 2017).
a. Self-neglect was not considered as part of the definition, given that it lacks an interpersonal component and is not usually considered under EA in the UK (McDermott, 2010).
2) Contain information about:
i. Several key variables: (a) abuse type(s), (b) victim’s gender, (c) victim-perpetrator relationship, and (d) enquirer’s identity (victim vs. non-victim)
ii. Help-seeking (e.g., barriers, facilitators, sources of help) from the perspective of the victims and non-victim enquirers.

### Materials

A data collection tool was developed to gather the data needed for the purposes of this study. The first author focused on the free texts and used the data collection tool to gather case characteristics. This tool was created based on an extensive review of previous literature, with a particular focus on a systematic review of victim vulnerability and perpetrator risk factors in EA (Storey, 2020)—to gather relevant victim and perpetrator characteristics—and a review of victims’ help-seeking behavior (Fraga Dominguez et al., 2021b), as help-seeking was the focus of the project. The coding scheme recorded primarily nominal variables, some with categories (e.g., victim’s gender), but many coded dichotomously as present or absent (e.g., victim’s mental health problems). The variables recorded and relevant to the present study are included in Table 1. The data collection tool consisted of several sections, relating to characteristics of the (1) Enquirer, (2) Victim, (3) Abuse, (4) Victim-perpetrator relationship, (5) Perpetrator, and and (6) Help-seeking. The working definitions of several of the variables relating to the victim, perpetrator, and abuse sections are provided in Supplementary File 1.

**Table 1.** Data Collection Variables.

Section	Categorical variables with categories	Numerical variables
Enquiry and enquirer	• Multiple victims and perpetrators: Yes/no	• If multiple perpetrators: Number of victims and perpetrators
	○ If multiple victims/perpetrators: relationship between them	
	• Enquirer type: victim versus non-victim	
	○ If non-victim	
	▪ Relationship with the victim: Family member, friend, neighbor, partner, professional, acquaintance, other	
	▪ Relationship with the perpetrator: Family member, friend, neighbor, partner, professional, acquaintance, stranger, other	
	• Source of signposting (i.e., how they heard about the service): Age UK, Silverline, internet, previous contact, other.^a,b^	
	• Source of enquiry: Telephone, email, letter, other^b^	
	• Nation of enquiry: England, Scotland, Wales, Northern Ireland^b^	
Victim	• Demographic characteristics	• Demographic characteristics: age^b,c^
	○ Gender: male, female, other	
	○ Race/ethnicity^b^	
	○ Relationship status: Single, married, living with partner, widowed, divorced	
	○ Deceased: Yes/no	
	• Vulnerability factors (all yes/no): Physical health problems, physical disability, intellectual disability, mental health problems, dementia, substance abuse problems, previously victimized	
	• Mental capacity (all yes/no): lacks capacity according to enquirer, assessed by professional as lacking capacity.	
Perpetrator	• Demographic characteristics: Same as victim with the exception of “deceased”, which was not coded	• Demographic characteristics: age^b,c^
	• Risk factors (all yes/no): Same as victim, with the addition of antisocial attitudes	
Victim-perpetrator relationship	• Specific relationship^d^: Family member, professional, friend, neighbor, other	
	○ If family member: Adult child, partner, grandchild, stepchild, sibling, nephew/niece, other, unspecified	
	• Victim and perpetrator co-habitation (yes/no)	
	• Victim’s dependency on the perpetrator: Yes/no.	
	○ If yes, type (yes/no): For care, socially or emotionally, financially, for housing, other	
	• Perpetrator’s power of attorney status (yes/no)	
	• Perpetrator’s dependency on the victim: Same as victim	
Abuse	• Abuse type (all yes/no): Financial, neglect, physical, psychological, sexual	• If poly-victimization: Number of abuse types co-occurring
	• Poly-victimization (two or more types of abuse co-occurring): Yes/no	
	• Abuse location^d,e^: victim’s home, care home/nursing home, hospital, sheltered accommodation, other	
	• Other abuse characteristics (all yes/no)	
	○ Frequency and duration: Abuse ongoing, one-time incident, chronicity (described as long-standing, lasting more than 6 months)	
	○ Abusive behaviors: use of isolation techniques, use of threats.	
	○ Other abuse characteristics: Long-standing intimate-partner violence (before the victim was aged 60), bi-directionality of abuse (i.e., both perpetrator and victim are abusive towards each other), substantiation of the abuse	
	• Impact on victims: Financial, physical health, psychological health	

^a^Age UK is an organization working with older people in the UK. The Silverline is a free helpline for older people, their families, and friends, open 24 hours a day every day.

^b^Information about this variable was obtained from Hourglass’ database.

^c^The age of victims and perpetrators was only used when this information was unavailable in the free text. In many cases, data about ages were present in both Hourglass’ database fields and on the free text, thus Intraclass Correlation Coefficient (ICC_1_) (Bartko, 1966) (mixed effects, absolute agreement) was calculated. The results were .986 (victim’s age) and .998 (perpetrator’s age) suggesting excellent agreement (Koo & Li, 2016). This provides confidence in using Hourglass’ variables.

^d^Denotes the variables for which categories were exclusive.

^e^If there were multiple locations, the location where most of the abuse occurred was coded.

#### Cases with multiple victims and perpetrators

In cases with multiple victims and perpetrators, the number of victims/perpetrators was recorded, and the researchers gathered information about up to two victims and/or perpetrators. Information about any further victims or perpetrators was not recorded because these cases were uncommon, and, in most cases with more than two perpetrators, information about those perpetrators was limited (e.g., being described as “relatives” or “care home workers”). The primary victims and perpetrators coded as the “main victim” and “main perpetrator” were the ones that were the center of the enquiry. This was defined as the older adult (or perpetrator) who was described as suffering (or perpetrating) most of the abuse, who was mentioned first, or who had the closest relationship with the perpetrator (or victim), in this order. For the secondary victim or perpetrator, basic information (i.e., gender) is presented herein.

### Final Sample

After applying inclusion criteria, out of the 2,538 entries in the system, 1,623 (64%) met inclusion criteria. The main reason for excluding 915 cases was that they did not meet the EA definition (*n* = 207, 23%) or that there was not enough information to conclude whether the case was EA (*n* = 192, 21%). Other reasons for exclusion were: no information about key variables (*n* = 135, 15%), victim younger than 60 (*n* = 110, 12%), enquiring about a telephone number (*n* = 75, 8%), description of systemic abuse (*n* = 50, 6%), suspicion of abuse (*n* = 39, 4%), test case (i.e., database system testing) or duplicate (*n* = 32, 4%), repeat enquiry (*n* = 30, 3%), other (*n* = 28, 3%), and no information about help-seeking (*n* = 17, 2%).

### Inter-Rater Reliability

To ensure the coding was performed reliably, a research assistant (RA) independently coded 254 cases (10% of the original sample of records; *N* = 2,538). The RA signed a confidentiality agreement with the charity before receiving a sample of 254 fully anonymized cases from the first author, randomly generated from the original sample. The first author trained the RA on the data collection tool and several practice cases were coded together to ensure consistency. The RA started coding in February 2019 and completed it in July 2019.

Inter-rater reliability was calculated for these cases using Cohen’s Kappa for categorical variables and Intraclass Correlation Coefficient (two-way, mixed methods, and absolute agreement) for continuous variables (e.g., age). Percent agreement was calculated when Cohen’s Kappa could not be calculated because the variable was a constant. Cohen’s Kappa results ranged from .68 to .87, indicating good to very good agreement (Altman, 1999), and ICC_1_ ranged from .80 to 1, indicating good to excellent agreement (Koo & Li, 2016). See Supplementary Material 2 for the average inter-rater reliability results by category.

### Data Analysis

Data were entered into SPSS version 21, which was used to generate descriptive statistics for the variables with pre-identified categories. Due to lack of research relating to victims who lack mental capacity, and institutional abuse cases, the frequencies of several characteristics are reported for those cases specifically, and chi-square tests of independence were used to test whether those characteristics were significantly related to victim’s mental capacity and abuse location (community vs. institutional).

## Results

### General Sample Characteristics

#### Enquiry Details

Most enquiries were made via telephone (*n* = 1,550, 96%), although some were email and letter (*n* = 73, 4%) enquiries. The helpline recorded the way enquirers heard about the service in 756 cases (47%). Most of those enquirers had heard about the helpline online (*n* = 438, 58%), followed by Age UK (*n* = 109, 14%). Most enquiries were made from England (*n* = 1,270, 78%), with a minority from Scotland (*n* = 52, 3%), Wales (*n* = 51, 3%), and Northern Ireland (*n* = 21, 1%) (0.4% outside of the UK, 14% unknown).

### Enquirers’ Characteristics

Most enquirers discussed the abuse of someone else (*n* = 1,434, 88%), with 12% being self-reported victimization cases. Most third-party callers were relatives of the victim (*n* = 1,077, 75%) and the perpetrator (*n* = 791, 55%), and female (*n* = 1,020, 71%). Friends (*n* = 93, 6%), neighbors (*n* = 81, 6%), and acquaintances (*n* = 78, 5%) were other common enquirers. Finally, professionals were the enquirers in 67 cases (5%).

### Number of Victims and Perpetrators

Among the 1,623 cases, a minority mentioned multiple victims (*n* = 119, 7%), with an average of 2.0 victims (*sd* = 0.1) in those cases. More cases involved multiple perpetrators (*n* = 363, 22%), with an average of 2.1 perpetrators (*sd* = 0.5) in said cases. The most common relationship between victims was that of partners/spouses (*n* = 101, 85%). Multiple perpetrators were most commonly siblings (*n* = 82, 23%), partners/spouses (*n* = 80, 22%), or colleagues (*n* = 60, 17%; e.g., in a care/nursing home). Another co-perpetrator relationship was a parent and an adult child (*n* = 49, 13%), and several relationships were unknown (*n* = 24, 7%). The descriptive statistics that follow focus on the primary victim and perpetrator.

### Victim and Perpetrator Characteristics

Characteristics for the main victim and main perpetrator can be found in Table 2. Primary victims were predominantly female and aged 80.9 years on average (*SD* = 8.9). In the sample including 119 secondary victims (*n* = 1,742), victims were still predominantly female (*n* = 1,149, 66%). Primary perpetrators were most commonly male and aged on average 51.9 years (*SD* = 17.3). When the sample also included secondary perpetrators (*n* = 1,986), these were also most commonly male (*n* = 833, 51%; 339 cases unknown).

**Table 2.** Primary Victim and Primary Perpetrator Characteristics.

	Victim		Perpetrator	
	Cases		Cases	
	*n*	%	*n*	%
Gender^a^				
Female	1093	67.3	682	48.7
Male	529	32.6	719	51.3
Relationship status^a^				
Single	24	4.8	43	11.8
Married or living with partner	315	62.5	87	79.0
Widowed or divorced	165	32.8	33	9.1
Deceased	77	4.7		
Any vulnerability or risk factor^b^	781	48.1	507	31.2
Physical health problems	351	21.6	21	1.3
Physical disability	121	7.5	7	0.4
Intellectual disability	9	0.6	6	0.4
Mental health problems	105	6.5	80	4.9
Dementia	320	19.7	7	0.4
Lacks capacity according to enquirer	104	6.4		
Assessed by professional as lacking capacity	83	5.1		
Lacks capacity according to enquirer and/or assessment	108	6.7		
Antisocial attitudes			352	21.7
Substance abuse problems	12	0.7	91	5.6
Previously victimized	39	2.4	10	0.6

^a^Percentages are provided for valid cases.

^b^“Any vulnerability or risk factor” indicates cases where victims or perpetrators have any of the following: physical health problems, physical disability, intellectual disability, mental health problems, dementia, substance abuse problems, or previous victimization. For perpetrators, antisocial attitudes are also included.

Victims’ race/ethnicity was obtained through Hourglass’ records; however, it was only recorded in 181 cases (11%). In these cases, victims were predominantly White-British (*n* = 126, 70%). Other victims were Asian of different backgrounds (*n* = 32, 18%), Black of African or Caribbean background (*n* = 11, 6%), and White-Irish or White of any other background (*n* = 11, 6%).

### Victim-Perpetrator Relationship

The victim-perpetrator relationships and other related variables can be found in Table 3. The perpetrators were primarily relatives, and there was frequent co-habitation. Victims were dependent on the perpetrators for care, and the perpetrator was the victim’s main caregiver in more than half of those cases (*n* = 229, 53%).

**Table 3.** Relationship of the Perpetrator With the Victim.

		Cases	
		*n*	%
Victim-Perpetrator relationship	Family member	1193	73.5
	Adult child	760	46.8
	Partner	188	11.6
	Grandchild	59	3.6
	Nephew/niece	46	2.8
	Sibling	30	1.8
	Stepchild	13	0.8
	Other family member	68	4.2
	Family member unspecified	29	1.8
	Professional	206	12.7
	Friend	137	8.4
	Neighbor	54	3.3
	Other	33	2.0
Victim and perpetrator co-habitation		505	31.1
Victim’s dependency on the perpetrator	Any	630	38.8
	For care	436	26.9
	Perpetrator is victim’s power of attorney	160	9.9
	Socially or emotionally	130	8.0
Perpetrator’s dependency on the victim	Any	84	5.2
	Housing	50	3.1
	Financially	40	2.5

### Abuse Characteristics

The abuse type and characteristics can be found in Table 4. The abuse reported was predominantly financial or psychological, and in more than a third of cases there was co-occurrence/poly-victimization. Among these cases, the average number of types of abuse suffered was 2.2 (*sd* = .4) and the abuse types most likely to co-occur were financial and psychological. The abuse types most likely to co-occur with others were physical (*n* = 155/196, 79%) and psychological (*n* = 552/803, 69%). Neglect co-occurred in 202 out of 369 cases (55%) and financial in 499 out of 994 cases (50%). The most likely to occur in isolation was sexual abuse, co-occurring in 11 out of 27 cases (41%). Most cases were perpetrated in the victim’s own home and ongoing at the time of the enquiry, with only a few one-time incidents. Finally, most cases (*n* = 1,256, 78%) mentioned at least one type of impact for the victim, frequently financial.

**Table 4.** Abuse Characteristics.

	Cases	
	*n*	%
Abuse type		
Financial	994	61.2
Psychological	803	49.5
Neglect	369	22.7
Physical	196	12.1
Sexual	27	1.7
Abuse poly-victimization		
Any co-occurrence	653	40.2
Financial and psychological^a^	413	63.2
Psychological and neglect^a^	123	18.8
Psychological and physical^a^	120	18.4
Financial and neglect^a^	119	18.2
Abuse location^b^		
Victim’s home	1211	80.3
Care home/nursing home	174	11.5
Hospital	52	3.6
Sheltered accommodation	37	2.5
Other	32	2.1
Other abuse characteristics		
Abuse ongoing^b^	1420	89.0
One-time incident^b^	59	3.8
Chronicity	364	22.4
Perpetrator isolation	186	11.5
Use of threats	50	3.1
Substantiated	14	0.9
Long-standing intimate partner violence	18	1.1
Bi-directional^c^	7	0.4
Impact on victims		
Financial	746	46.0
Psychological	488	30.1
Physical	412	25.4

^a^Percentages are provided for cases of poly-victimization.

^b^Percentages are provided for valid cases.

^c^In these cases, the person who was framed as the main recipient of abuse by the enquirer and the helpline, was coded as the victim.

### Characteristics by Mental Capacity Status and Abuse Location

In cases where the victim lacked capacity, as compared to cases where the victim had capacity, victims were more likely to have a vulnerability, including dementia (see Table 5). They were more likely to suffer financial abuse and be abused in institutions, but less likely to suffer from psychological abuse or poly-victimization or be abused by multiple perpetrators.

**Table 5.** Case Characteristics by Victim’s Mental Capacity and Abuse Location.

		Mental capacity (*n* = 1515)		No mental capacity (*n* = 108)		χ^2^	Community (*n* = 1211)		Institutional (*n* = 174)		χ^2^
		*n*	%	*n*	%		*n*	%	*n*	%	
Multiple victims		112	7.4	7	6.5		96	7.9	10	5.7	
Multiple perpetrators		350	23.1	13	12.0	7.109^**^	264	21.8	51	29.3	4.884^*^
Victim characteristics	Female	1019	67.3	74	68.5		826	68.3	115	66.1	
	Male	495	32.7	34	31.5		384	31.7	59	33.9	
	Deceased	69	4.6	8	7.4		33	2.7	15	8.6	15.807^***^
	Any vulnerability factor	704	46.5	77	71.3	24.892^***^	567	46.8	106	60.9	12.106^***^
	Dementia	260	17.2	60	55.6	93.882^***^	207	17.1	70	40.2	50.900^***^
	Lacks capacity						56	4.6	32	18.4	48.458^***^
Perpetrator characteristics	Female	633	48.1	49	57.0		530	47.7	53	56.4	
	Male	682	51.9	37	43.0		581	52.3	41	43.6	
	Any risk factor	478	31.6	29	26.9		435	35.9	26	14.9	30.151^***^
Victim-perpetrator relationship	Family	1114	73.5	79	73.1		951	78.5	75	43.1	270.335^***^
	Professional	189	12.5	17	15.7		80	6.6	87	50.0	
	Other	212	14.0	12	11.1		180	14.9	12	6.9	
Victim’s dependency on perpetrator		581	38.3	49	45.4		465	38.4	104	59.8	28.710^***^
Abuse type	Financial	918	60.6	76	70.4	4.059^*^	748	61.8	78	44.8	18.137^***^
	Psychological	773	51.0	30	27.8	21.792^***^	658	54.3	54	31.0	33.067^***^
	Neglect	340	22.4	29	26.9		264	21.8	70	40.2	28.238^***^
	Physical	189	12.5	7	6.5		154	12.7	23	13.2	
	Sexual^a^						14	1.2	7	4.0	
Abuse co-occurrence		620	40.9	33	30.6	4.507^*^	529	43.7	53	30.5	10.919^***^
Abuse location	Community	1155	89.1	56	63.6	48.458^***^					
	Institution	142	10.9	32	36.4						

*Note.* Percentages are provided for valid cases.

Community cases refer to the abuse occurring at the victim’s own home and institutional cases to abuse occurring at a care or nursing home.

Chi-square statistics are provided for variables with significant findings.

Categories for variables were merged to avoid low frequencies.

^a^Frequencies omitted due to low frequencies.

*p < .05, **p < .01, ***p < .001

In comparison to abuse occurring in the community, institutional abuse was more likely to involve multiple perpetrators, victims who were deceased at the time of enquiry, and victims lacking mental capacity. Although victim vulnerability factors, including dementia, were more common, perpetrator risk factors were less common. The perpetrator was more likely to be a professional and the victim more likely to depend on the perpetrator. Nonetheless, more than a third of the perpetrators in institutional settings were relatives. Neglect was more common in institutional settings, while financial abuse, psychological abuse, and poly-victimization were more common in community settings.

## Discussion

The purpose of the present study was to describe EA cases reported to a UK helpline to provide updated knowledge of EA that can inform research and practice in the area. To the authors’ knowledge, this represents the description of the largest sample of EA cases in the UK. From the original 2,538 enquiries, researchers identified 1,623 EA cases. These cases primarily related to a female victim, suffering financial or psychological abuse—with abuse types co-occurring in over a third of cases—perpetrated by relatives, particularly their adult children.

### Enquiry Characteristics

The study provided information about the characteristics of enquirers to a national helpline, which can be helpful for this and similar national services offering advice (e.g., Age UK’s helpline). Victims self-reporting were a minority, potentially due to existing barriers to formally disclosing within this population, or the frequency of family perpetrators, as victims find these cases harder to report (Acierno et al., 2020; Fraga Dominguez et al., 2021b).

### Victims’ and Perpetrators’ Characteristics and Relationship

Victims were more often female, consistent with previous research (Bennett et al., 2000; Brijnath et al., 2021). In contrast with prevalence data in the UK (O’Keeffe et al., 2007), but consistent with other study types (Bennett et al., 2000; Brijnath et al., 2021), perpetrator gender was almost equally distributed. Victims’ vulnerabilities and perpetrators’ risk factors were common, particularly physical health problems or dementia in victims—consistent with research on vulnerability factors Storey, (2020)—and antisocial attitudes, mental health/substance abuse problems in perpetrators. Adding to victims’ needs, impact resulting from the abuse was reported in 78% of cases. These findings underscore the need for healthcare professionals’ detection and intervention, as victims and perpetrators might be in contact with healthcare services (Pillemer et al., 2016), as well as the need for follow up and victim support services after disclosure. Institutional abuse victims and those lacking mental capacity were more likely to have vulnerabilities, particularly dementia, suggesting increased needs in these populations.

Perpetrators were often related to the victim, particularly adult children, consistent with previous research (Bennett et al., 2000; Brijnath et al., 2021; Weissberger et al., 2020). Partners were the next most common perpetrator amongst relatives, in contrast with O’Keeffe et al.’s (2007) prevalence study. Older adults victimized by partners might contact different helplines or services that focus on intimate partner violence. Unsurprisingly, institutional abuse victims were more likely to be abused by professionals than victims in the community. Importantly, 7% of cases involved multiple victims and 22% multiple perpetrators. Studies rarely report whether cases concern single or multiple victims/perpetrators; however, those that do have reported similar rates of multiple perpetrators (Lachs & Berman, 2011; Weissberger et al., 2020). The existence of multiple perpetrators may be associated with severity and increased negative impact for victims (Ramsey-Klawsnik, 2017). Multiple victims were often spouses and the relationship between perpetrators was more diverse. This information contributes to our understanding of the relational dynamics in EA (Brijnath et al., 2021).

### Abuse Characteristics and Location

Regarding the abuse types reported, and consistent with previous research (Bennett et al., 2000; Brijnath et al., 2021; Weissberger et al., 2020; Yon et al., 2017), financial and psychological abuse were the most common. Although studies differ in whether financial—as in the present study—or psychological abuse is most common, these two types are understood to be the most prevalent (Yon et al., 2017). Differences between samples can be attributed to varied definitions and a bias in the type of cases more likely to be included due to sampling (Weissberger et al., 2020). Rates of poly-victimization were high, with co-occurrence of multiple abuse types identified in 40% of cases, and consistent with research by Bennett et al. (2000) and Brijnath et al. (2021). In line with Brijnath et al. (2021), Weissberger et al. (2020), and Williams et al. (2020), the abuse most likely to co-occur was physical, followed closely by psychological. The most common combination of two abuse types was financial and psychological, followed by psychological and neglect, and psychological and physical, consistent with Brijnath et al.’s (2021) research. Finally, institutional abuse victims may be particularly at risk of neglect and victims lacking capacity at risk of financial abuse.

Although recent systematic reviews have indicated that EA is common in both community and institutional settings (Yon et al., 2017, 2018), the present study found that over 80% of reported cases occurred in the community, and this percentage was higher than in the previously reported helpline analysis (Bennett et al., 2000). There are several possible reasons for this contrast. First, institutional EA may be more likely to be reported to other services that are specific to these settings (e.g., the Care Quality Commission in England) which either had not been formed in 2000 or were less accessible. Second, due to higher likelihood of vulnerability factors—as seen in the current study—and potentially less oversight by relatives, these cases may be more likely to remain unreported and undetected (Bennett et al., 2000; Fraga Dominguez et al., 2021b). Finally, institutional abuse cases might be dealt with internally, which could also contribute to less external reporting (Grant & Benedet, 2016).

### Limitations and Strengths

This study is limited in several ways; primarily because of the nature of secondary data gathered outside of the research team, affected by self-selection bias and the lack of case substantiation. Helpline staff do not regularly ask a set of questions and the information for some variables (e.g., perpetrator characteristics) is more likely to be missing. Although the helpline is not a source of formal reporting or investigation and enquirers can and often remain anonymous, the sample may still be affected by under-reporting. These and other limitations have been discussed in detail by Weissberger et al. (2020). Nevertheless, this organization’s helpline was chosen due to its strengths in terms of national recognition and special focus on EA (Podnieks et al., 2010). Furthermore, the sample characteristics in terms of EA types, poly-victimization, and victim-perpetrator relationship were largely consistent with research using both similar and prevalence data (Bennett et al., 2000; Brijnath et al., 2021; Lachs & Berman, 2011; Weissberger et al., 2020; Williams et al., 2020).

There are also some limitations relating to the diversity of the sample. Although, among the cases where race/ethnicity was recorded, the proportion of minority groups was higher than estimated in the older UK population (ONS, n. d.), the cases with this information were too few to determine the representativeness of the sample. Due to lack of information in the free texts, it is not possible to know how representative the sample is of different socioeconomic backgrounds and sexual or gender minorities. Although there are concerns in gathering such information in a regular helpline context, where the priority is advising enquirers, and when enquirers may not be comfortable sharing these data, an effort to improve records where possible will help services understand whether they are reaching the diversity of the UK population.

Despite these limitations, this study also has many strengths. The sample encompasses both abuse occurring in the community and institutional abuse, and presents characteristics by location, an uncommon feature in EA research (Fraga Dominguez et al., 2021b). Additionally, because enquirers are both victims and third parties, cases where the victim may have cognitive limitations and/or communication difficulties, or where they may be unable to self-advocate, are still represented (Brijnath et al., 2021; O’Keeffe et al., 2007). This is supported by the frequency of cases where victims had various needs, including dementia, and cases where victims were described as lacking mental capacity. The findings also expand on areas unexplored in previous UK research, such as the existence of multiple victims and perpetrators, specific patterns of poly-victimization, abusive behaviors, and relationship dynamics.

### Implications for Research, Policy, and Practice

This study suggests, along with previous research, that financial abuse and psychological abuse are common types, and as such a priority in terms of prevention and intervention. However, sexual abuse is likely under-reported, due to shame and assumptions around older age and sex (Goldblatt et al., 2022), stressing the need to study this abuse type using different data sources (Weissberger et al., 2020). The poly-victimization rates in the study reinforce professionals’ need to assess multiple abuse types even if presented with just one type, particularly because risk factors may differ across abuse types, including co-occurrence, requiring different intervention approaches (Fraga Dominguez et al., 2022; Jackson & Hafemeister, 2011; Weissberger et al., 2020). Researchers should aim to advance our understanding of poly-victimization, by investigating the way in which abuse types co-occur (e.g., if one type of abuse facilitates others; Ramsey-Klawsnik, 2017). A further understanding of poly-victimization can inform awareness campaigns and training for professionals, so that victims, the public, and practitioners are able to recognize first signs of abuse. Other pieces of evidence arising from the study findings that should inform awareness campaigns are that EA may be perpetrated by multiple individuals and that older people in institutions can be at risk of EA by someone other than professionals.

Several findings contrasted with previous prevalence research in the UK, suggesting that each data source is biased in different ways and represents a different group of victims, perpetrators, and abuse types. Thus, researchers must gather data in different ways; official reports to police or adult safeguarding, population surveys, and analysis of helplines (Weissberger et al., 2020) are all useful sources. Further research in the UK population using other sources is necessary to complement the findings of the current study. In this research, more information should be obtained about perpetrators’ needs, so that this knowledge can be used to manage and prevent EA cases (Storey, 2020). The current findings may underestimate the frequency of perpetrator needs, as the enquiries’ focus was on the victims’ circumstances. Researchers should also aim to provide data over longer time periods, to be able to study trends (Brijnath et al., 2021), including whether reported cases are increasing as the population continues to age (WHO, 2021). Such studies would help to understand the changing nature of abuse perpetrated against older adults, which can inform future policy, including resource allocation.

## Conclusions

The present study aimed to provide an update on the characteristics of EA cases reported to a national UK helpline. The study identified that the most common abuse types were financial and psychological, and that poly-victimization happened in more than a third of the cases. Although this updated knowledge is helpful for guiding practice, future research is needed, ideally examining a longer time period, in order to study trends in the abuse reported and the characteristics and victims and perpetrators.

## Supplemental Material

Supplemental Material—Characterizing Elder Abuse in the UK: A Description of Cases Reported to a National Helpline Click here for additional data file. Supplemental Material for Characterizing Elder Abuse in the UK: A Description of Cases Reported to a National Helpline by Silvia Fraga Dominguez, Jennifer E. Storey, and Emily Glorney in Journal of Applied Gerontology
