# Supplementary material for: Characterizing Elder Abuse in the UK: A Description of Cases Reported to a National Helpline
Source: J Appl Gerontol. 2022 Jun 28;41(11):2392–403. doi: 10.1177/07334648221109513 (PMC9561799; doi:10.1177/07334648221109513)
Supplement: Supplemental Material—Characterizing Elder Abuse in the UK: A Description of Cases Reported to a National Helpline [file sj-pdf-1-jag-10.1177_07334648221109513.pdf]

Supplementary Material 1

Table 1

Definition of Data Collection Variables

Section	Variable and Definition
Victim and perpetrator	• Physical health problems: poor physical health or medical problems (Storey, 2020).<br>• Physical disability: “limitation on a person’s physical functioning or mobility” (De la Torre-Luque et al., 2017, p. 423).<br>• Intellectual disability: “disability characterized by significant limitations both in intellectual functioning (reasoning, learning, problem solving) and in adaptive behavior, which covers a range of everyday social and practical skills” (American Association on Intellectual and Developmental Disabilities, 2020, para. 1).<br>• Mental health problems: diminished psychological health and mental health problems, e.g., depression and anxiety (Storey, 2020).<br>• Dementia: “a syndrome in which there is deterioration in memory, thinking, behavior, and the ability to perform everyday activities” (WHO, 2020, para. 1).<br>• Substance abuse problems: problems related to the use of illegal substances or misuse of legal substances, such as alcohol or prescribed medication (Storey, 2020).<br>• Previously victimized: previous abuse experienced or witnessed, other than the current episode of EA (for victims) or during childhood or adolescence (for perpetrators) (Storey, 2020)<br>• Antisocial attitudes: not taking responsibility for behavior, and antisocial behavior, such as a history of criminal or violent behavior (Storey, 2020). For example, if it was reported in the enquiry that the perpetrator was violent or had committed a crime against someone other than the victim.
Abuse types and behaviors	• Financial abuse: the unauthorized and improper use of funds, property or any resources of an older person. Examples: stealing of money, possessions or property, the use of fraud, or the misuse of power of attorney.<br>• Psychological abuse: the (…) use of threats, humiliation, bullying, swearing and other verbal conduct, and/or any other form of mental

cruelty that results in mental or physical distress. Examples: insulting the victim, calling the victim names, threatening the victim, undermining or belittling the victim, and preventing the victim from seeing others that they care about. Actions considered as coercive control (Barlow et al., 2020).

- Physical abuse: the non-accidental infliction of physical force that results in a bodily injury, pain, or impairment. Examples: slapping the victim, grabbing, pushing or shoving the victim, threatening the victim with a knife, gun, or other weapon, locking the victim in their room, or giving the victim drugs or too much medicine to control them.
- Sexual abuse: direct or indirect involvement in sexual activity without consent. Examples: talking to the victim in a sexual way that makes them feel uncomfortable, touching them in a sexual way against their will.
- Neglect: repeated deprivation of assistance needed by the older person for important activities of daily living. Neglect may be intentional or unintentional (e.g., because a caregiver cannot cope or is not getting sufficient help).
- Use of isolation: for example, the perpetrator prevents the victim from seeing other members of the family or even talking to them. In more extreme cases, they may have taken away the victim's telephone to prevent them from making calls.
- Use of threats: utterances or behavior that threatens physical, psychological, or social harm, such as threatening statements.

Note. The definitions for the abuse types used can be found in O'Keeffe et al. (2007, pp. 18-19). The primary researcher and the research assistant (RA) used these definitions as a reference during coding, along with examples extracted from the website of Hourglass (at the time, Action on Elder Abuse) for the different types of abuse. The same exact examples are no longer available due to changes in the organization's website; thus, they are included herein for reference

Mental health problems, substance abuse problems, and antisocial attitudes were all coded separately, because these have been separately identified as risk factors or vulnerability factors.

References in the table that are not included in the manuscript:

American Association on Intellectual and Developmental Disabilities (2020). *Frequently asked questions on intellectual disability.* https://www.aaidd.org/intellectual-disability/definition/faqs-on-intellectual-disability

Barlow, C., Johnson, K., Walklate, S., & Humphreys, L. (2020). Putting coercive control into practice: Problems and possibilities. *The British Journal of Criminology*, *60*(1), 160-179. https://doi.org/10.1093/bjc/azz041

De la Torre-Luque, A., Valero-Aguayo, L., & de la Rubia-Cuestas, E. J. (2017). Visuospatial orientation learning through virtual reality for people with severe disability. *International Journal of Disability, Development and Education*, *64*(4), 420-435. https://doi.org/10.1080/1034912X.2016.1274022

World Health Organization. (2020, September 21). *Dementia: Key facts*. https://www.who.int/news-room/fact-sheets/detail/dementia

Supplementary Material 2

Table 2

Inter-Rater Reliability Average Results by Category

Section	Kappa	ICC_1	Percent agreement
Case and enquirer characteristics	.82	.99	
Victim characteristics	.82	1	100
Perpetrator characteristics	.87	.99	99.8
Victim-perpetrator relationship characteristics	.70		
Abuse characteristics	.74	.80	98.3
Previous help-seeking and facilitators	.68		100
Barriers	.74		99.6
Advice	.78		

Note. Percent agreement was calculated when Cohen's Kappa could not be calculated because the variable was a constant.
